# Epidemiological characteristics of imported influenza viruses at Shanghai entry–exit ports from 2017 to 2024

**DOI:** 10.3389/fpubh.2026.1780983

**Published:** 2026-03-17

**Authors:** Xinyi Ma, Ye Lu, Shiwei Yu, Zaijiong Yi, Chunli Hu, Liming Xue, Zilong Zhang, Zhengan Tian, Shenwei Li

**Affiliations:** 1 School of Public Health, Nanjing Medical University, Nanjing, China; 2 Shanghai International Travel Healthcare Center (Shanghai Customs Port Clinic), Shanghai, China

**Keywords:** epidemiological characteristics, imported influenza virus, lineage change, port surveillance, post-COVID-19 recovery

## Abstract

**Objectives:**

To characterize long-term epidemiological patterns of imported influenza at Shanghai entry–exit ports from 2017 to 2024 and to compare changes before and after the COVID-19 pandemic, thereby providing a basis for optimizing cross-border surveillance strategies.

**Methods:**

Surveillance data were collected from febrile inbound travelers identified by infrared temperature screening (≥37.3 °C) at major Shanghai ports during 2017–2019 and 2023–2024. Throat swabs were tested using real-time RT-qPCR for influenza A and B viruses. Positivity rates, seasonality, demographic characteristics, geographic origins, and viral type distributions were compared between pre- and post-COVID-19 periods using appropriate statistical tests.

**Results:**

Among 33,118 febrile inbound travelers screened, 6,163 influenza cases were confirmed, yielding an overall positivity rate of 18.61%. No significant difference was observed between the pre- and post-COVID-19 periods (18.94% vs. 18.52%, *p* = 0.34), despite a marked increase in case numbers following travel resumption. Imported influenza exhibited a persistent bimodal seasonal pattern, with winter–spring peaks largely associated with Northern Hemisphere sources and summer peaks primarily driven by Southern Hemisphere imports. The geographic origin of cases shifted significantly, with the Western Pacific Region accounting for 66.2% of cases in 2024, while contributions from the Americas declined (*p* < 0.05). Adults aged 25–59 years increasingly predominated, and the proportion of travelers aged ≥60 years rose over time. Influenza A viruses remained dominant throughout the study period, accounting for 93.3% of cases in 2023, while influenza B showed a partial rebound in 2024.

**Conclusion:**

Imported influenza at Shanghai entry–exit ports rebounded rapidly after COVID-19, with stable transmission intensity but notable shifts in geographic origin, age composition, and viral distribution. The persistence of bimodal seasonality and increasing concentration of cases from the Western Pacific Region highlight the need to transition from fixed seasonal screening toward dynamic, risk-based port surveillance with emphasis on high-traffic routes and adult travelers.

## 1 Introduction

Influenza viruses (IFVs), single-stranded RNA viruses of the family *Orthomyxoviridae*, are characterized by their capacity for antigenic drift and shift, resulting in both seasonal epidemics and global pandemics (1–6). Globally, approximately one billion people are infected with influenza each year, leading to 290,000–650,000 respiratory-related deaths (accounting for 8.2% of such deaths) and an estimated economic loss of approximately 500 billion USD (7–10). With the expansion of international travel and trade, IFVs can spread across borders within 24 h (11, 12). Surveillance of imported cases at international entry–exit ports serves as a frontline defense for detecting emerging variants and preventing outbreaks.

Under the framework of the International Health Regulations (IHR, 2005) and national infectious disease control laws in China, port health authorities are mandated to conduct surveillance for imported infectious diseases and monitor viral variation. Previous studies (13, 14) have shown that imported IFVs detected at entry–exit ports display diverse subtype distributions and high genetic similarity to globally circulating strains, reflecting frequent cross-border transmission. Although most detected cases are considered imported infections, their potential contribution to local influenza dynamics remains an important public health concern.

Shanghai, a major aviation hub in the Asia–Pacific region, received over 18.08 million international arrivals in 2024. As the largest international entry–exit port in China, Shanghai handles the highest volume of inbound travelers and the most extensive network of international flights, covering a wide range of source countries and regions. This high connectivity and diversity of travel origins make Shanghai a highly representative setting for monitoring imported influenza and assessing cross-border transmission risks. Its port-based fever screening system plays a sentinel role in identifying imported influenza cases (15). During the COVID-19 pandemic (2020–2022), stringent control measures markedly reduced influenza activity, potentially altering its seasonality and subtype dynamics (16). In the post-COVID-19 period (2023–2024), international travel has rapidly resumed, yet empirical evidence remains limited regarding whether the epidemiological characteristics of imported influenza have undergone structural changes. Current research has primarily focused on domestic influenza patterns (17, 18), with limited systematic analyses comparing long-term surveillance data on imported influenza before and after the COVID-19 pandemic.

This study analyzes influenza surveillance data from Shanghai entry–exit ports during 2017–2019 (pre-COVID-19 period) and 2023–2024 (post-COVID-19 period), comparing the epidemiological features of imported influenza cases across the two periods, thereby providing evidence to optimize cross-border infectious disease surveillance and early-warning strategies.

## 2 Methods

### 2.1 Study population

Travelers entering Shanghai through international entry–exit ports were screened using infrared thermoscanners. Those with abnormal forehead temperature (≥37.3 °C) underwent confirmatory manual temperature measurement, epidemiological investigation, assessment of influenza-like illness (ILI) symptoms, and throat swab collection by trained quarantine officers. All febrile inbound travelers during 2017–2019 and 2023–2024 were laboratory tested, and only RT-qPCR–confirmed influenza cases were included in the analyses.

### 2.2 Data collection and processing

Data were extracted from the Customs Health Quarantine Information System. Because travel and personal information is systematically recorded through the customs system at entry, demographic and travel-related data were complete for all screened travelers. After sample collection, laboratory testing was performed to exclude non-influenza respiratory infections, and only RT-qPCR–confirmed influenza cases were retained for analysis. During data processing, duplicate records resulting from multiple entries by the same traveler within 1 week were identified and removed, with only the first entry retained. Routine influenza surveillance at Shanghai entry–exit ports was suspended during the COVID-19 pandemic, resulting in an interruption of data collection between 2020 and 2022.

### 2.3 Influenza virus nucleic acid testing

A 200-μL aliquot of each sample was processed using the viral nucleic acid extraction kit from Jiangsu Bioperfectus Technologies Co., Ltd. (magnetic-bead method), according to the manufacturer’s instructions. Influenza A/B detection was performed using Real-time quantitative reverse transcription PCR (RT-qPCR) kits from Jiangsu Bioperfectus Technologies Co., Ltd., Shanghai ZJ Bio-Tech Co., Ltd., and Shanghai BioGerm Medical Technology Co., Ltd. Results were interpreted according to the criteria for result interpretation specified in the manufacturer’s instructions.

### 2.4 Calculation of positivity rate

The influenza positivity rate was defined as the proportion of laboratory-confirmed influenza cases among febrile inbound travelers who underwent throat swab sampling at entry–exit ports. The influenza positivity rate was calculated as:


$$\text{Positivity rate}(\%)=\frac{\begin{array}{l} \text{Number of}\;\mathrm{RT}-\text{qPCR}–\text{confirmed}\;\\ \text{influenza}-\text{positive cases} \end{array}}{\begin{array}{l} \text{Total number of samples tested}\\ \text{within the same time period} \end{array}}\times 100$$

This definition was applied to calculate both annual positivity rates (2017, 2018, 2019, 2023, and 2024) and weekly positivity rates used for seasonality analyses.

### 2.5 Seasonality analysis

Based on imported influenza surveillance data collected at Shanghai entry–exit ports during two study periods (2017–2019 and 2023–2024), weekly influenza activity was assessed using weekly positivity rates, as defined in Section 2.4. To account for differences in influenza seasonality across regions, imported cases were grouped according to the hemisphere of their country of origin (Northern Hemisphere and Southern Hemisphere), and weekly positivity rates were calculated separately for each group. Line charts were constructed with ISO 8601 calendar week on the *x*-axis and weekly positivity rate on the primary *y*-axis to illustrate temporal patterns of influenza activity. In addition, the weekly number of samples tested was plotted on a secondary *y*-axis to provide contextual information on weekly sampling volume.

Because imported influenza cases originate from multiple countries with distinct epidemic timings and are influenced by international travel patterns, their seasonal trends may not follow the typical winter–spring pattern observed in the Northern Hemisphere. Therefore, epidemic periods were identified using a dynamic threshold method commonly applied by the U.S. Centers for Disease Control and Prevention (CDC), based on weekly positivity rate fluctuations (19, 20): when the weekly positive rate exceeds the annual average for three consecutive weeks, this is defined as the commencement of the epidemic season for that year; subsequently, if the curve continues to rise and exhibits a distinct peak, the peak center is defined as the point of highest relative proportion. The epidemic peak period for that year is then delineated by extending this point to the weeks before and after when the positive rate falls below the annual average. When the positive rate remains below the annual average for three consecutive weeks and the curve shows a clear decline, this is regarded as the end of the epidemic season or an intermittent trough period.

### 2.6 Demographic analysis

For all influenza-positive cases confirmed by RT-qPCR, demographic characteristics were analyzed. Sex was classified as male and female. Age groups were classified as 0–4, 5–14, 15–24, 25–59, and ≥60 years (21). Country of origin was defined according to the six regions: African Region, Region of the Americas, Eastern Mediterranean Region, European Region, South-East Asia Region, and Western Pacific Region as defined by World Health Organization (WHO).

### 2.7 Statistical analysis

All analyses were performed using SPSS version 26.0 (IBM Corp., Armonk, NY, USA). Categorical variables (sex, age group, virus type, and region of origin) were summarized as counts and percentages, and continuous variables (e.g., age) were reported as medians due to non-normal distributions.

Between-group comparisons were conducted using the chi-square test (or Fisher’s exact test when expected counts were <5), the Mann–Whitney U test for two-group comparisons, and the Kruskal–Wallis H test for multiple groups. Differences in positivity rates between periods were assessed using a two-sample Z test.

Multivariable logistic regression was used to evaluate independent associations between study period and demographic variables (sex, age group, and region of origin), and a period × age group interaction term was included to assess structural shifts in demographic composition.

## 3 Results

### 3.1 Annual positivity rates

During the two study periods (2017–2019 and 2023–2024), a total of 95.58 million inbound travelers entered Shanghai entry–exit ports. Among them, 33,118 febrile travelers were screened, yielding 6,163 influenza-positive cases (overall positivity rate: 18.61%) (Table 1). Annual rates were 19.26% (2017), 15.26% (2018), 22.38% (2019), 17.93% (2023), and 18.82% (2024). Pre- and post-COVID-19 period rates were comparable (18.94% vs. 18.52%), with no significant difference (*Z* = 0.95, *p* = 0.34). Although the number of positive cases increased in 2023–2024 (1,533.5 vs. 1,032.0 cases per year), the overall positivity rate remained stable.

**Table 1:** Annual variation in inbound population and positivity rate of imported influenza at Shanghai entry–exit ports, 2017–2024.

Year	Number of inbound travelers at Shanghai entry–exit ports (million)	Number of throat swabs tested	Number of influenza-positive cases	Positivity rate (%)
2017	21.80	5,821	1,121	19.26
2018	23.10	5,360	818	15.26
2019	22.80	5,169	1,157	22.38
2023	9.80	5,777	1,036	17.93
2024	18.08	10,791	2,031	18.82
Total	95.58	33,118	6,163	18.61
2017–2019	67.70	16,350	3,096	18.94
2023–2024	27.88	16,568	3,067	18.52

### 3.2 Seasonal analysis

**Figure 1:**
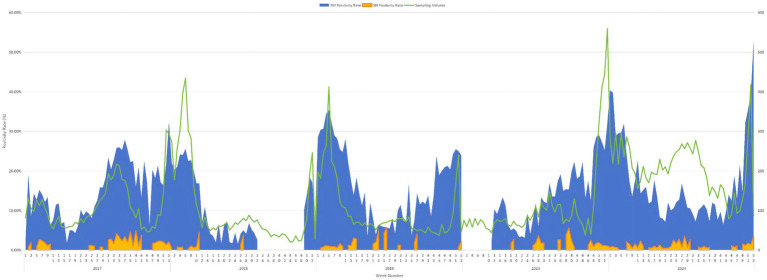
Seasonal distribution of imported influenza cases and differences in origin between the Northern and Southern Hemispheres, 2017–2024.

Imported influenza activity showed clear seasonal variability across study years (Figure 1), based on weekly positivity rates by hemispheric origin. Weekly sampling volume (number of throat swabs tested) is shown on the secondary *y*-axis to aid interpretation of temporal trends. In 2017, a summer sub-peak occurred during weeks 31–35 with simultaneous rises in both hemispheres, followed by the main winter–spring peak from week 51 to week 9 of 2018. In 2018, a summer sub-peak was observed in weeks 30–33, mainly driven by Southern Hemisphere imports. The 2018–2019 winter–spring peak spanned weeks 52 of 2018 to 8 of 2019, with a summer sub-peak in weeks 27–32 of 2019. The 2019 winter–spring peak began in week 51. After surveillance resumed, imported cases in 2023 rose from week 26, forming a summer peak in weeks 26–33, predominantly from the Southern Hemisphere, followed by a winter secondary peak from week 50 to week 4 of 2024. The 2024 summer peak occurred during weeks 28–36, again mainly driven by Southern Hemisphere imports.

### 3.3 Demographic features

#### 3.3.1 Sex distribution

Males consistently represented a higher proportion (mean annual proportion: 55.6% ± 1.8%), with stable sex ratios across years (Table 2). No significant difference was found between pre- and post-COVID-19 periods (*χ*^2^ = 1.44, *p* = 0.23).

**Table 2:** Annual gender distribution of imported influenza cases, 2017–2024.

Year	Male	Female
2017	625 (55.8%)	496 (44.2%)
2018	434 (53.1%)	384 (46.9%)
2019	669 (57.8%)	488 (42.2%)
2023	576 (55.6%)	460 (44.4%)
2024	1,163 (57.3%)	868 (42.7%)

#### 3.3.2 Age distribution

Age distribution changed significantly over time (Table 3). Prior to the COVID-19 pandemic, children and adolescents accounted for a substantial proportion of imported cases, whereas during the post-pandemic period the proportion of adults increased markedly. Individuals aged 25–59 years became the dominant group, accounting for more than 60% of cases in 2024, while the proportion of children aged 0–4 years declined to below 2%.

**Table 3:** Dynamics of age distribution among imported influenza cases, 2017–2024.

Age group	2017	2018	2019	2023	2024	*aOR* (95% *CI*)	*p* value
0–4 y	93 (8.3%)	104 (12.7%)	64 (5.5%)	20 (1.9%)	32 (1.6%)	1.00 (ref)	—
5–14 y	163 (14.5%)	192 (23.5%)	283 (24.5%)	111 (10.7%)	277 (13.6%)	3.01 (2.18–4.15)	<0.001
15–24 y	144 (12.8%)	80 (9.8%)	96 (8.3%)	178 (17.2%)	191 (9.4%)	5.69 (4.08–7.93)	<0.001
25–59 y	538 (48.0%)	396 (48.4%)	615 (53.1%)	612 (59.1%)	1,268 (62.4%)	5.94 (4.38–8.04)	<0.001
≥60 y	183 (16.3%)	46 (5.6%)	99 (8.6%)	115 (11.1%)	263 (13.0%)	5.64 (4.05–7.86)	<0.001
Median age	33	27	30	31	35	—	—

*aORs* were estimated using multivariable logistic regression with study period [post-COVID-19 period (2023–2024) vs. pre-COVID-19 period (2017–2019)] as the dependent variable. The model included sex and WHO region of origin as covariates. A period × age-group interaction term was added to assess effect modification. 95% *CIs* are shown.

Multivariable logistic regression analysis demonstrated that age group remained independently associated with the study period. Compared with children aged 0–4 years, cases aged 5–14 years (*aOR* = 3.01), 15–24 years (*aOR* = 5.69), 25–59 years (*aOR* = 5.94), and ≥60 years (*aOR* = 5.64) were significantly more likely to be detected in the post-COVID-19 period (all *p* < 0.001).

Interaction analysis further revealed a significant interaction between study period and age group (*p* < 0.001), indicating that the post-pandemic resurgence of imported influenza was characterized by a structural shift toward adult age groups rather than proportional increases across all ages.

#### 3.3.3 Geographic origin

The geographic distribution of imported influenza cases also changed significantly over time (Table 4). The Western Pacific Region remained the predominant source throughout the study period. However, the contribution from the Eastern Mediterranean Region increased after the pandemic, whereas the proportion of cases originating from the Region of the Americas declined.

**Table 4:** Geographic distribution of imported influenza cases, 2017–2024.

Region	2017	2018	2019	2023	2024	*aOR* (95% *CI*)	*p* value
Eastern Mediterranean	30 (2.7%)	12 (1.5%)	31 (2.7%)	48 (4.6%)	72 (3.5%)	1.58 (1.14–2.18)	0.006
South-East Asia Region	219 (19.5%)	103 (12.6%)	154 (13.3%)	157 (15.2%)	328 (16.1%)	1.00 (ref)	—
Africa	14 (1.2%)	4 (0.5%)	5 (0.4%)	1 (0.1%)	19 (0.9%)	0.92 (0.49–1.72)	0.796
Americas	88 (7.9%)	50 (6.1%)	75 (6.5%)	68 (6.6%)	64 (3.2%)	0.67 (0.52–0.87)	0.002
Europe	129 (11.5%)	84 (10.3%)	98 (8.5%)	92 (8.9%)	203 (10.0%)	0.92 (0.75–1.13)	0.419
Western Pacific	641 (57.2%)	565 (69.1%)	794 (68.6%)	670 (64.7%)	1,345 (66.2%)	1.05 (0.91–1.21)	0.545

*aORs* were estimated using multivariable logistic regression with study period [post-COVID-19 period (2023–2024) vs. pre-COVID-19 period (2017–2019)] as the dependent variable. The model adjusted for sex and age group. 95% *CIs* are shown. The reference category was Southeast Asia.

After adjustment for sex and age group, regional distribution remained independently associated with the study period. Cases from the Eastern Mediterranean Region were more likely to be detected in the post-pandemic period (*aOR* = 1.58, *p* = 0.006), whereas those from the Region of the Americas were significantly less frequent (*aOR* = 0.67, *p* = 0.002).

Age distribution differed significantly across WHO regions, both in median age and age-group composition, and modest differences in sex distribution were also observed (all *p* < 0.01) (Table 5).

**Table 5:** Demographic characteristics of imported influenza cases by WHO region, Shanghai entry–exit ports, 2017–2024.

Region	Median age (IQR)	0–4 y	5–14 y	15–24 y	25–59 y	≥60 y	Male (%)
Eastern Mediterranean	39 (26–53)	1.6	7.3	11.9	64.3	15	61.1
South-East Asia Region	37 (25–54)	1.7	8.8	9.7	59.4	20.5	58.7
Europe	35 (22–49)	2.9	11	11	59.1	16.1	55.5
Americas	30 (13–46)	8.4	15.2	13.3	46.4	16.7	59.1
Western Pacific	31 (16–43)	6	18.2	11.2	51.2	13.3	55.2
Africa	33 (12–45)	14	20.9	4.7	46.5	14	76.7

### 3.4 Influenza A and B distribution

**Figure 2:**
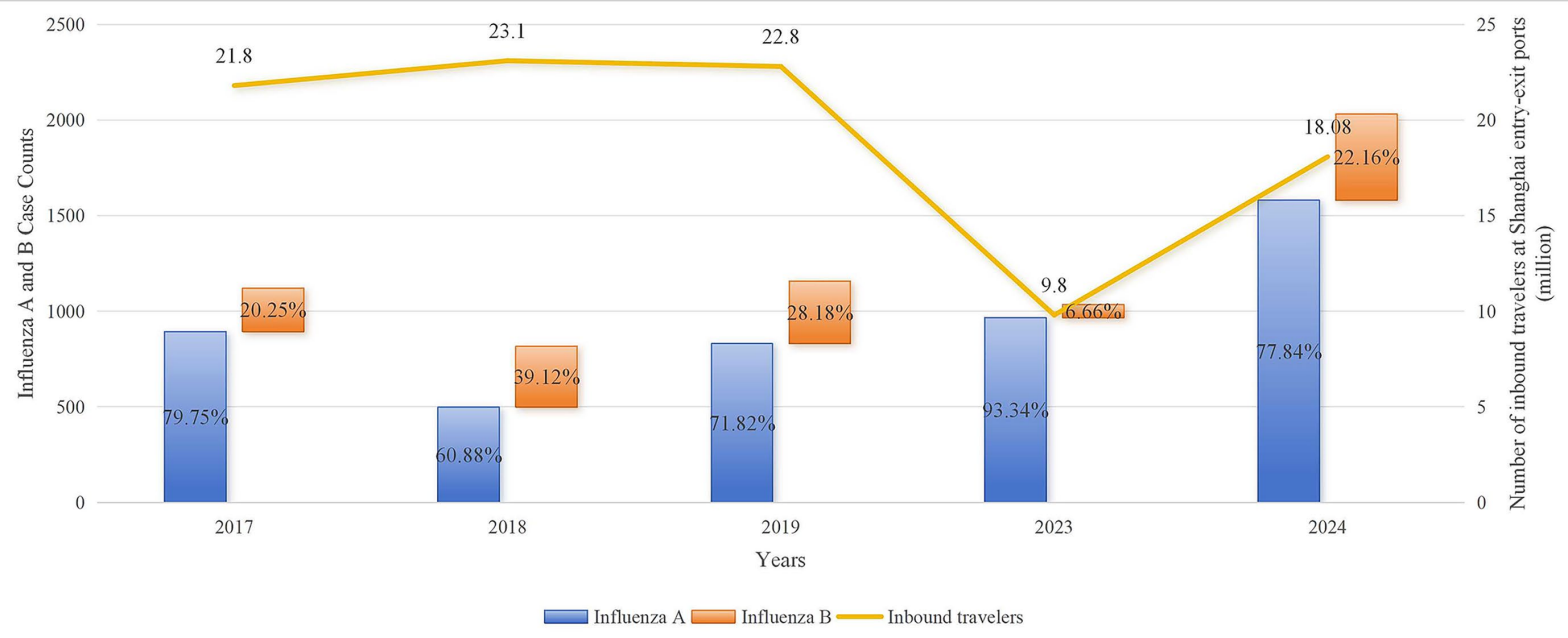
Annual distribution of influenza A and B viruses among imported cases, 2017–2024.

Influenza A viruses accounted for a higher proportion of imported influenza cases across all study years (Figure 2). The annual proportion of influenza A ranged from 60.88 to 93.34%, peaking in 2023. Influenza B accounted for 6.66% of cases in 2023 but rebounded to 22.16% in 2024, approaching pre-COVID-19 period levels.

To further evaluate whether the post-pandemic period independently influenced virus type composition, univariable and multivariable logistic regression analyses were performed. In univariable analysis, the study period was not associated with virus type distribution (*OR* ≈ 1.00, *p* = 0.99). After adjustment for age group and region of origin, the period effect remained non-significant (adjusted *OR* ≈ 1.00, *p* = 0.999), indicating that the relative proportions of influenza A and B remained stable between the pre- and post-COVID-19 periods.

No significant interaction between study period and hemisphere was observed (*p* = 0.999), suggesting consistent subtype distribution patterns across hemispheric regions. Influenza A was more frequently detected among older adults, whereas a lower proportion of influenza A was observed among cases originating from the Western Pacific Region.

## 4 Discussion

### 4.1 Strategic value and mechanism of port-based surveillance

Influenza activity at Shanghai entry–exit ports showed a marked resurgence in 2023–2024, with imported case numbers returning to or exceeding pre-COVID-19 period levels. Notably, despite the increase in detected cases, the overall positivity rate remained comparable to that observed during 2017–2019, suggesting that the underlying risk of influenza importation has stabilized rather than intensified.

This pattern is closely associated with strengthened port public health measures implemented in China during the post-COVID-19 period, particularly the enhanced surveillance of imported infectious diseases (22). Following the pandemic, entry–exit port surveillance placed greater emphasis on respiratory infections, with an expanded scope of fever screening among inbound travelers and the implementation of targeted testing strategies (23). These measures increased the likelihood of identifying influenza cases, including those with mild or transient febrile symptoms, without indicating an actual increase in transmission intensity. Within this framework, port-based surveillance operates through a multi-layered, risk-based mechanism. Global epidemic trends are continuously assessed to identify high-risk regions, based on which monitoring is selectively intensified through non-invasive infrared temperature screening (24). Fever screening functions as an initial, rapid filter suitable for large-scale cross-border populations, while travelers flagged at this stage subsequently undergo focused epidemiological investigation and laboratory confirmation.

Our findings indicate that this fever-based screening approach remains applicable in both routine surveillance (pre-COVID-19) and periods of intensified monitoring (post-COVID-19). Importantly, this strategy is not limited to influenza but represents a foundational methodology for the early identification of a broad range of infectious diseases at international borders. By integrating dynamic risk assessment with convenient primary screening and targeted follow-up investigation, this surveillance framework offers practical implications for scalable and evaluable global port health surveillance.

### 4.2 Seasonal variation and bimodal characteristics of imported influenza

This study identified a bimodal temporal pattern in imported influenza cases, with a primary peak occurring during calendar weeks 51–10 and a secondary peak during weeks 28–32. These peaks were predominantly associated with cases originating from the Northern Hemisphere and Southern Hemisphere, respectively, together contributing to year-round fluctuations in importation risk. Similar dual-peak temporal patterns have been consistently reported in port-based and international surveillance studies from Europe, South-East Asia Region, and major global travel hubs, reflecting the asynchronous epidemic cycles across different geographic regions rather than uniform climatic seasonality (25–29). Importantly, such temporal patterns were well documented prior to the COVID-19 pandemic, indicating that the post-pandemic observations in Shanghai represent a resumption of established global influenza transmission dynamics rather than a novel shift.

At a broad temporal scale, imported influenza activity showed general concordance with local influenza surveillance in Shanghai (20, 30), with overlapping peak periods in corresponding calendar weeks. In certain years, modest temporal leads were observed between imported and local case curves, suggesting that port-based surveillance may offer supplementary temporal context for interpreting influenza activity, rather than acting as a direct or independent predictor of local transmission dynamics.

### 4.3 Age distribution of imported cases

Adults (25–59 years) and older travelers (≥60 years) constituted the majority of imported influenza cases. Although the overall median age did not differ significantly between periods, multivariable analysis indicated that adult and older travelers were significantly more likely to be identified in the post-pandemic period, independent of region of origin. Moreover, the significant interaction between study period and age group suggests a structural demographic shift toward adult travelers rather than proportional increases across all age groups.

In contrast, local influenza surveillance in Shanghai indicates that cases are predominantly reported among children (38.5%) (31, 32), whereas imported influenza cases mainly affected adults (62.4%). This discrepancy likely reflects differences in the populations under surveillance: international travelers are overwhelmingly adults, while pediatric populations constitute the primary affected group in community-based influenza transmission.

### 4.4 Regional concentration and control challenges

Case origins became increasingly concentrated in the Western Pacific Region (66.2% by 2024), while contributions from the Americas declined markedly (3.2%). This trend aligns with earlier recovery of Asia–Pacific flight networks and regional economic integration (33). Short-haul travel from South-East Asia Region, in particular, has become a major driver of importations (34). Surveillance strategies should therefore be optimized by tailoring monitoring to high-risk routes and regions, with enhanced risk-based screening. Stratified analyses further revealed significant regional differences in age distribution, suggesting that region-specific traveler profiles may contribute to the observed demographic patterns.

### 4.5 Compositional changes in influenza A and B viruses

The composition of imported IFVs showed pronounced annual fluctuations. In the post-COVID-19 period (2023–2024), influenza A viruses demonstrated absolute dominance, accounting for 93.34% of imported influenza cases at Shanghai entry–exit ports in 2023. This finding aligns closely with the global trend where influenza A viruses constituted approximately 98.8% of cases during the same period (35, 36). However, after adjustment for age group and region of origin, no independent period effect on influenza A/B distribution was observed. This suggests that the dominance of influenza A reflects broader global circulation patterns rather than a structural post-pandemic shift in virus ecology. This dominance likely reflects multiple factors. First, the accumulation of “immunity debt” due to prolonged non-pharmaceutical interventions (NPIs)—such as mask-wearing and social distancing—reduced natural infections and vaccination coverage, weakening population immunity (37, 38). Second, influenza A inherently exhibits higher transmissibility than type B, enabling faster spread once restrictions were lifted (39–42). Finally, the resumption of social activities and international travel facilitated cross-border transmission and exposure, accelerating the resurgence and renewed predominance of influenza A. Consistent with previous port-based and international surveillance, imported influenza viruses were dominated by influenza A, especially A(H3N2), and showed high genetic similarity to globally circulating strains (43).

### 4.6 Implications for port surveillance and public health practice

This study highlights the continued strategic value of port-based surveillance in settings with high volumes of international travel. Fever screening remains an economical and operationally feasible first-line tool for large-scale entry–exit populations, enabling rapid identification of potentially infectious travelers without disrupting passenger flow. As an initial screening measure, it is most effective when integrated with dynamic risk assessment, focused epidemiological investigation, and laboratory confirmation.

To further improve detection sensitivity, port surveillance may be strengthened by incorporating enhanced risk stratification based on travel origin, targeted testing strategies, and improved health communication to encourage proactive symptom reporting among travelers from high-risk regions. The surveillance framework implemented in Shanghai provides a practical and scalable model that can be adapted to other international entry–exit ports.

### 4.7 Study limitations

This study was conducted at Shanghai entry–exit ports and may not fully represent all ports across China, given regional differences in travel patterns and surveillance capacity. In addition, the interruption of surveillance during 2020–2022 precluded assessment of transitional trends and may limit interpretation of temporal continuity. Furthermore, reliance on fever-based screening may lead to under-detection of a proportion of afebrile or asymptomatic infections. Due to the lack of country-specific inbound travel volume data, regional differences should be interpreted cautiously, as fluctuations in travel intensity may partially influence geographic distribution patterns.

## 5 Conclusion

Using surveillance data from Shanghai entry–exit ports (2017–2019 and 2023–2024), this study demonstrates that imported influenza rebounded rapidly after the COVID-19 pandemic (2023–2024) with increased detection volume but a stable overall positivity rate. Imported cases maintained a persistent seasonal bimodal pattern—summer peaks driven primarily by Southern Hemisphere sources and winter peaks by Northern Hemisphere sources—while geographic origins shifted toward the Western Pacific Region. Age distribution moved toward adults and older travelers, and influenza A dominated in 2023 with influenza B partially rebounding in 2024. These results support transitioning from fixed seasonal screening toward dynamic, risk-based port surveillance focused on high-traffic routes and vulnerable traveler groups to strengthen early warning and cross-border control.

## Data Availability

The original contributions presented in the study are included in the article/supplementary material, further inquiries can be directed to the corresponding author.
